# Diffuse large B-cell lymphoma-leg type masquerading as venous eczema

**DOI:** 10.1016/j.jdcr.2024.11.028

**Published:** 2024-11-30

**Authors:** Harsha Moolchandani, Bo Liu, Joel Cunningham, Joy Staniforth, Ben Marriott, Azaharry Yaakub

**Affiliations:** a Department of Dermatology, Norfolk and Norwich University Hospitals NHS Foundation trust, Norwich, United Kingdom; b Department of Haematology, Norfolk and Norwich University Hospitals NHS Foundation trust, Norwich, United Kingdom; c Haematopathology and Oncology Diagnostic Services (HODS), Cambridge University Hospitals NHS Foundation Trust, Cambridge, United Kingdom; d Department of Histopathology, Norfolk and Norwich University Hospitals NHS Foundation trust, Norwich, United Kingdom

**Keywords:** cellulitis, chronic venous insufficiency, cutaneous B-cell lymphoma, histopathology, immunohistochemistry, localized radiotherapy, venous eczema

## Summary

Primary cutaneous diffuse large B-cell lymphoma-leg type (PCDLBCL-LT), is a rare high-grade form of primary cutaneous lymphoma. It often presents as red to violaceous nodules or plaques on lower limbs. We report a case of PCDLBCL-LT presenting initially as cellulitis. The skin changes did not respond to oral and intravenous antibiotics and management for venous eczema was subsequently commenced. The patient then developed 2 large deep subcutaneous masses and core biopsy confirmed a diagnosis of a mature high-grade B-cell lymphoma. Review of the patient’s history revealed a prior diagnosis of high-grade B-cell lymphoma 22 years previously. This case demonstrates that PCDLBCL-LT can present with clinical signs in keeping with a diagnosis of cellulitis or venous eczema. In the context of progressive nodular skin changes biopsy should always be considered, particularly when the patient has a prior history of a hematologic malignancy.

## Report

A 74-year-old woman presented with a 4-month history of a red, hot swollen lower portion of the right leg without any systemic upset or biochemistry abnormality. She received 5 courses of antibiotics for presumed cellulitis without improvement.

Her past medical history included right knee and hip replacements, previous trauma to the right leg after road traffic accident that required complex reconstruction and was complicated with right leg cellulitis. Additionally, 22 years ago, she was treated for localized DLBCL on the forehead, which achieved remission with 3 cycles of cyclophosphamide, doxorubicin, vincristine, and prednisone chemotherapy and radiotherapy.

**Fig 1:**
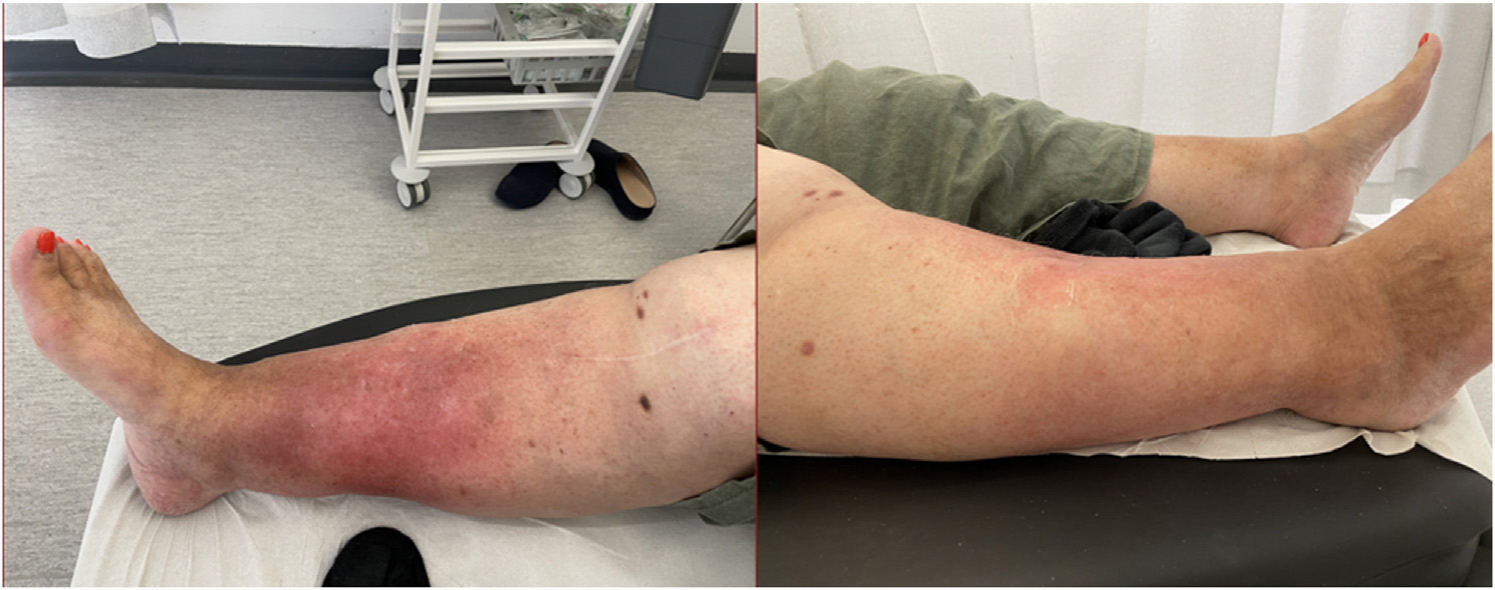
Both lower limbs showing hemosiderin discoloration, a knee replacement scar on anterior aspect of the right knee. Right medial, anterior lower portion of the leg showing erythema with edema and wooden hard texture. Pedal pulses were palpable.

Based on clinical findings of chronic venous insufficiency changes over both lower portion of the legs, she was managed for acute lipodermatosclerosis (Fig 1).

**Fig 2:**
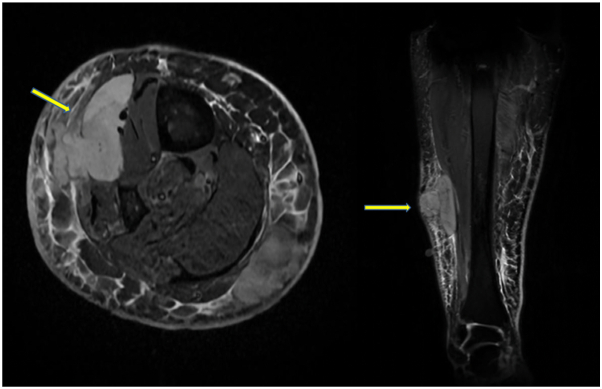
Magnetic resonance imaging scan of the lower portion of the right leg, demonstrating multiple soft tissue nodules within the subcutaneous tissue and muscles, spanning the facial planes, with the largest one measuring 9 cm. Heterogeneous intermediate signal on T2 image raised the suspicion of lymphoma.

A week later, she represented to the emergency department for rapidly enlarging mass lesions in the right calf in a circumferential distribution. A magnetic resonance imaging scan was arranged (Fig 2).

**Fig 3:**
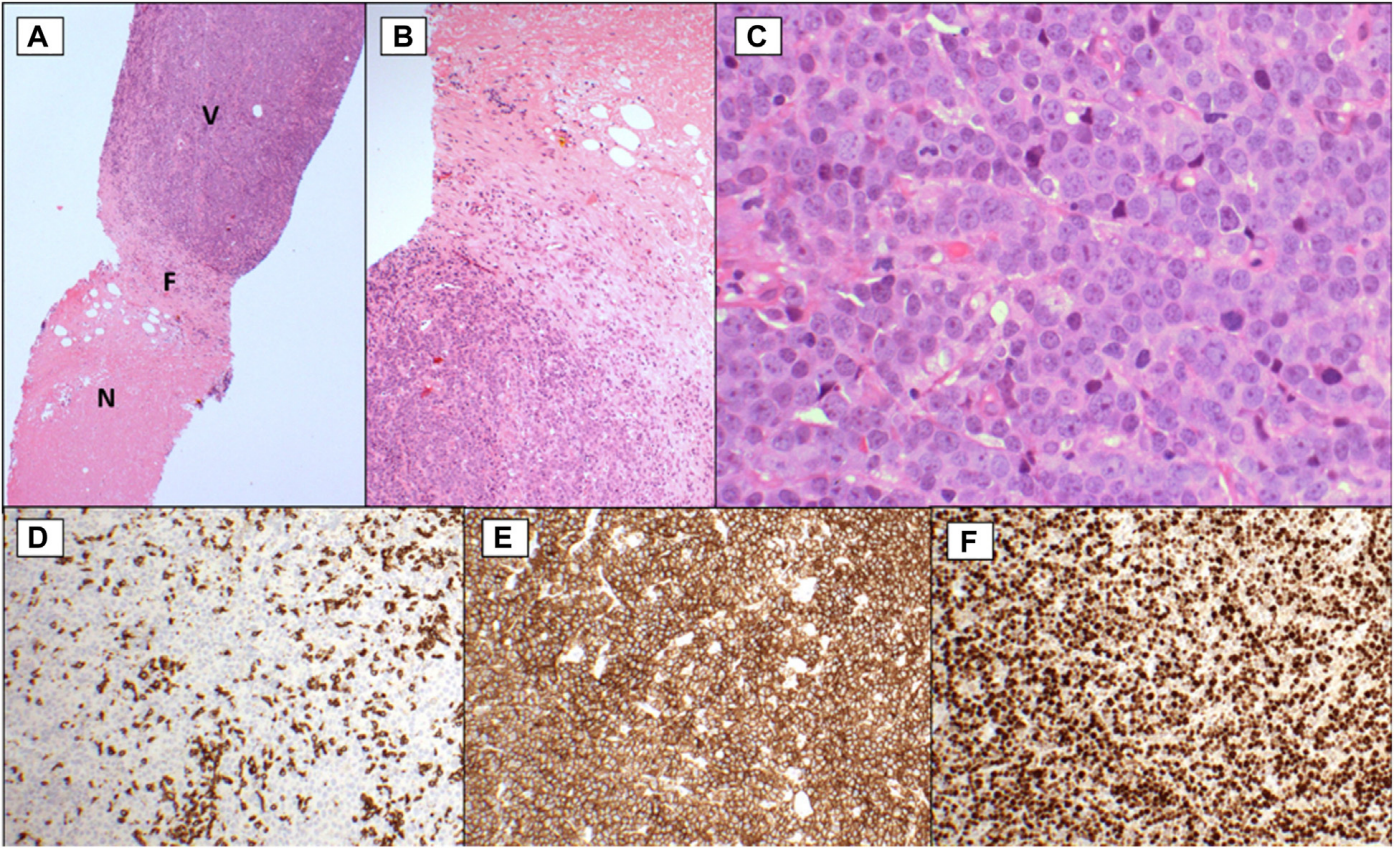
Core biopsy from mass lesion, right calf (**A**), viable tumor (V) with a necrotic segment (N) and intervening fibrosis (F). **B,** Center of image (**A**). **C,** The viable tumor cells; morphologically these are large atypical lymphoid cells, with rounded nuclei and multiple nucleoli. **D,** Positive (brown) staining in scattered small T cells but not the tumor cells. **E,** Positive staining in the tumor cells consistent with B-cell origin. **F,** The high proliferation index of the neoplastic B cells. (**A** and **C,** Hematoxylin-eosin stain; **D,** CD3 immunostain; **E,** CD20 immunostain; **F,** MIB1 immunostain; original magnifications: **A,** ×40; **B** and **D-F,** ×100; **C,** ×400.)

Based on the magnetic resonance imaging findings, 2 core biopsies were taken from the mass lesions, which revealed a partly necrotic high-grade B-cell lymphoma (Fig 3). On immunohistochemistry, the neoplastic cells expressed CD20, PAX, BCL6, MUM1, BCL2, and c-Myc, and had a high MIB1 proliferation index of 60% to 80%. CD10 expression was weak and variable; using the Hans algorithm, tumor in one of the biopsies met criteria for classification as germinal center immunophenotype and the other as nongerminal center. The neoplastic B cells did not express CD3, CD34, TdT, CD5, Cyclin D1, SOX-11, CD30, CD23, or CD21, and were negative for Epstein-Barr virus-encoded small RNAs by in situ hybridization. Fluorescent in-situ hybridisation showed no evidence of MYC, BCL2, or BCL6 gene rearrangements. While the majority of PCDLBCL-LT are negative for CD10, weak positivity may be seen in a minority of cases; the histologic differential diagnosis was secondary involvement by systemic DLBCL, NOS.^1^ Material from the original forehead skin biopsy was not retrieved for comparative B-cell clonality testing as after multi disciplinary team discussion this was not of clinical utility.

The patient was referred to the hematology team for further management. A staging PET/CT revealed stage IV lymphoma with fluorodeoxyglucose-avid pelvic and right femoral nodes, foci in the bone marrow and intensely fluorodeoxyglucose avid deep soft tissue in the lower portion of the right leg. In the context of the clinical presentation, PCDLBCL-LT (with secondary lymph node involvement) was favored over systemic DLBCL, NOS (with secondary cutaneous involvement); however, in this setting the distinction would not carry significant therapeutic or prognostic differences.

In light of the prolonged period of remission after the patient’s initial diagnosis, 6 cycles of immunochemotherapy with rituximab, cyclophosphamide, doxorubicin, vincristine, and prednisone were planned. Because of the patient’s prior anthracycline dosage, doxorubicin was replaced with gemcitabine for the final cycle of treatment. End of treatment PET/CT imaging demonstrated partial metabolic response with 2 persistent fluorodeoxyglucose-avid subcutaneous nodules in the lower portion of the right leg. Radiotherapy was delivered to the right leg to consolidate the response, and subsequent PET/CT imaging has shown complete metabolic response. Close clinical monitoring continues, with chimeric antigen receptor T-cell therapy or bispecific antibody therapy to be considered if the patient experiences further relapse of disease.^2^

## Discussion

This is a case of PCDLBCL-LT presenting as chronic venous changes, with rapid progression of disease. PCDLBCL-LT typically presents as one or more large bluish-red nodules and plaques that grows quickly. A definitive diagnosis can only be made on a representative biopsy, preferably an excisional deep biopsy, in the correct clinical context. The quoted 5-year overall survival rate is between 36% and 45%,^3^ although with the advent of novel therapies this may significantly improve.

High grade B-cell lymphoma is treated with the aim to cure, although patients can relapse. Relapse is usually within the first few years. A relapse after 22 years is unusual and may represent progression of an underlying low-grade/indolent hematopoietic cell clone.

This case serves as a reminder that persistent skin changes that do not respond to treatment for infective or inflammatory conditions should be considered for biopsy, particularly in the context of nodular subcutaneous change, or in the presence of risk factors for lymphoid malignancy such as prior history of lymphoid or chronic immunosuppression.

## Conflicts of interest

None disclosed.
